# Measurement and decomposition of socioeconomic inequality in single and multimorbidity in older adults in China and Ghana: results from the WHO study on global AGEing and adult health (SAGE)

**DOI:** 10.1186/s12939-017-0578-y

**Published:** 2017-05-15

**Authors:** Rasha Kunna, Miguel San Sebastian, Jennifer Stewart Williams

**Affiliations:** 1 Epidemiology and Global Health, Department of Public Health and Clinical Medicine, Umeå University, SE-901 87 Umeå, Sweden; 2 Research Centre for Generational Health and Ageing Faculty of Health, University of Newcastle, New Lambton Heights, NSW 2305 Australia

**Keywords:** Inequalities, Inequities, Social determinants, Multi-morbidity, Wealth, Low-and middle-income countries, LMICs, Non-communicable diseases, NCDs

## Abstract

**Background:**

Globally people are living longer and enduring non-communicable diseases (NCDs) many of which co-occur as multimorbidity. Demographic and socioeconomic factors are determinants of inequalities and inequities in health. There is a need for country-specific evidence of NCD inequalities in developing countries where populations are ageing rapidly amid economic and social change. The study measures and decomposes socioeconomic inequality in single and multiple NCD morbidity in adults aged 50 and over in China and Ghana.

**Methods:**

The data source is the World Health Organization Study on Global AGEing and Adult Health (SAGE) Wave 1 (2007–2010). Nationally representative cross-sectional data collected from adults in China (*n* = 11,814) and Ghana (*n* = 4,050) are analysed. Country populations are ranked by a socioeconomic index based on ownership of household assets. The study uses a decomposed concentration index (CI) of single and multiple NCD morbidity (multimorbidity) covering arthritis, diabetes, angina, stroke, asthma, depression, chronic lung disease and hypertension. The CI quantifies the extent of overall inequality on each morbidity measure. The decomposition utilises a regression-based approach to examine individual contributions of demographic and socioeconomic factors, or determinants, to the overall inequality.

**Results:**

In China, the prevalence of single and multiple NCD morbidity was 64.7% and 53.4%, compared with 65.9% and 55.5% respectively in Ghana. Inequalities were significant and more highly concentrated among the poor in China (single morbidity CI = −0.0365: 95% CI = −0.0689,–0.0040; multimorbidity CI = −0.0801: 95% CI = −0.1233,-0.0368;). In Ghana inequalities were significant and more highly concentrated among the rich (single morbidity CI = 0.1182; 95% CI = 0.0697, 0.1668; multimorbidity CI = 0.1453: 95% CI = 0.0794, 0.2083). In China, rural residence contributed most to inequality in single morbidity (36.4%) and the wealth quintiles contributed most to inequality in multimorbidity (39.0%). In Ghana, the wealth quintiles contributed 24.5% to inequality in single morbidity and body mass index contributed 16.2% to the inequality in multimorbidity.

**Conclusions:**

The country comparison reflects different stages of economic development and social change in China and Ghana. More studies of this type are needed to inform policy-makers about the patterning of socioeconomic inequalities in health, particularly in developing countries undergoing rapid epidemiological and demographic transitions.

## Background

In all regions of the world people are living longer than previous generations [1]. Irrespective of socioeconomic development, the main causes of death and disability in older age groups are non-communicable diseases (NCDs) many of which occur in combination [2–4]. The co-occurrence of at least two chronic conditions in the same individuals is known as multimorbidity [5]. In low- and middle-income countries (LMICs) populations are ageing rapidly and the health of older people is further compromised by poverty and limited access to affordable healthcare [4, 6–13]. This paper examines inequalities in the socioeconomic distribution of single and multiple NCDs in adults aged 50 and over in China and Ghana.

Understanding how social, economic and demographic factors (or social determinants) impact on health and disability in older people is an important policy challenge [14]. In countries at all income levels the NCD burden is relatively higher in older age and amongst disadvantaged and marginalised individuals and groups, compared with the young and those with higher socioeconomic status (SES) [15, 16]. The well-documented inverse health and wealth gradient is augmented by a growing multimorbidity burden. This requires new ways of managing and treating ill health in older adults [4, 17, 18].

In high-income countries about two thirds of adults aged 65 and over have at least two chronic conditions, about 50% have at least three, and 20% endure five or more [5, 6, 19, 20]. Although data are scarce, it is estimated that about half of the older adults in LMICs experience multimorbidity [4]. In recent years epidemiologists have attempted to identify the clinical patterning of comorbid chronic conditions. A systematic literature review of studies on multimorbidity identified depression, hypertension and diabetes as the most prevalent co-occurring chronic diseases [7]. Another review of multimorbidity identified 63 groups comprising three or more diseases, the most common being cardiovascular and metabolic diseases, followed by mental health disorders and musculoskeletal conditions [21]. In national cross-sectional data from non-institutionalized adults aged 50 and over in Finland, Poland, Spain, China, Ghana, India, Mexico, Russia, and South Africa, hypertension, cataract, and arthritis were the most prevalent comorbid conditions [22]. A study of health-insured individuals aged 65 and over in Germany identified three broad multimorbidity patterns – cardiovascular/metabolic disorders, anxiety/depression disorders and pain, and neuropsychiatric disorders [23].

Multimorbidity has been associated with older age, female gender, behavioural risk factors such as smoking and obesity, low SES, and poor quality of life [5, 22, 24–27]. Multimorbidity leads to interactions and complications which impact negatively on health. The combined mortality risk from multi-morbid individuals exceeds the sum of individual risks [5, 24, 28]. In high-income countries, people with multimorbidity use health services more often and have longer hospital stays, more postoperative complications and higher costs of care. Nevertheless evidence-based guidelines to better inform the treatment of patients with multimorbidity are limited [21]. Understanding the socioeconomic patterning of multimorbidity is important for both clinical management and public health policy [21, 26, 29, 30].

Studies in high-income countries have reported that people of low SES are more likely to have multiple chronic conditions [23]. However, it is difficult to draw conclusions or generalize from evidence collected in LMICs because chronic conditions are under-diagnosed and under-treated [26, 31, 32]. A study of catastrophic health expenditure (CHE) and inequality in elderly households with chronic disease patterns in China, for example, showed that having two or more chronic diseases was associated with CHE in the more advantaged households that could afford treatment [13]. A study in India showed that NCD multimorbidity was associated with substantially higher healthcare utilisation and out-of-pocket expenditure but the researchers did not find evidence of association between SES and multimorbidity [26]. A secondary analysis of South Africa’s national household survey data (2005–2008) showed that multimorbidity was more prevalent among the poor [33].

Epidemiological research into socioeconomic inequalities in NCD multimorbidity in LMICs is urgently needed for policy and planning [14, 18, 26]. Governments in LMICs face major challenges in re-designing systems and re-organising infrastructure to achieve universal access to equitable, affordable and effective healthcare in line with the global post-2015 development agenda for universal health coverage [11, 34–37].

About 60% of people in the world live in Asia and 16% live in Sub-Saharan Africa (SSA) - two regions with heavily populated LMICs. The United Nations predicts that by 2050 these proportions will be 54% in Asia and 25% in SSA, and by 2100, 44% and 39% respectively [38]. The absolute numbers are therefore larger in Asia with China leading the world, but population growth is higher in SSA [39]. Rapid increases in NCD-related morbidity and mortality are occurring in SSA - the world’s youngest and also poorest region [40]. Only about 4.9% of the population in SSA is aged 60 and over, compared with 12% globally, yet there are twice as many adults aged 60 and over in SSA compared with northern Europe [41]. Older people with chronic conditions in SSA experience socioeconomic disadvantage in relation to their health and wealth [42–44].

Ghana is a low-income country on the west coast of the African continent with a population of about 27.5 million people. In Ghana increasing numbers of older adults are living with NCDs such as hypertension, diabetes, arthritis and angina and the rising prevalence of NCDs is shaped by socioeconomic and demographic factors [45, 46]. Poverty rates are high and a large proportion of the Ghanaian population does not have access to improved water and sanitation. The population aged 60 and over in Ghana is expected to double (from 5.7% to 10.4%) between 2015 and 2030, yet population growth and ageing is outpacing socioeconomic development [38, 47, 48].

In contrast China, a populous middle-income country of 1.38 billion people in Asia, is undergoing significant economic development and transformation from a lower-middle to an upper-middle income country. In 2015 the number of people aged 60 and over was 17% of the population and this proportion is projected to reach 45% of China’s 1.4 billion residents by 2030 [38, 49]. Over the past thirty years China’s economic growth has outpaced that in all other parts of the world. Although this has led to extraordinary increases in per capita income and declines in poverty rates, China has paid a price in terms of increased inequalities in wealth and health [50–52]. Despite the introduction of extended insurance coverage, the poor continue to have less access to healthcare than the rich [53, 54]. In China people who live to older age are likely to experience single and multiple chronic illnesses and have financial difficulty accessing the care that they need [55, 56].

This study uses comparable national data to investigate socioeconomic concentrations of single and multiple NCDs (i.e. multimorbidity) and their determinants in China and Ghana. The aims are to measure, compare and decompose socioeconomic inequality in single and multiple NCD morbidity in adults aged 50 and over. The work provides new evidence about socioeconomic inequalities in NCD morbidity in older adults in Asia and SSA.

## Methods

### Data source and study participants

The data source is the World Health Organization (WHO) Study on global AGEing and adult health (SAGE) Wave 1 (2007–2010). WHO-SAGE is a longitudinal study of nationally representative samples of adults aged 50 and over in China, Ghana, India, Mexico, Russia and South Africa. This major international study aims to address the gap in reliable and scientific evidence on ageing and adult health in six geographically diverse LMICs at different stages of economic and epidemiological transition.

WHO-SAGE used a multi-stage stratified random cluster sampling design to ensure representation of a range of living conditions and urban and rural localities in the country samples. Households were the final sampling units. The household questionnaire was completed by an individual on behalf of each selected household, and all persons aged 50 and over in these households were invited to complete the WHO-SAGE individual questionnaire.

Stratification in China was by provinces, after which eight rural counties and eight urban clusters were selected. The PSUs were created by dividing the rural counties into four townships and the urban clusters into four cities. In Ghana, the sample was stratified by administrative region and urban or rural location, resulting in 20 strata from which primary sampling units (PSUs) and households were selected. Face-to face interviews were conducted in China in 2008–2010 and in Ghana in 2008–2009. The response rates of those who completed interviews, among all eligible persons, were 93% in China and 80% in Ghana [57, 58].

This study uses individual-level data that are in the public domain. Every household and individual had a known non-zero probability of selection. WHO provides household- and person-level analysis weights with post-stratification adjustments for age and sex distributions and non-response. Further details of WHO-SAGE are available on the WHO-SAGE website http://www.who.int/healthinfo/sage/en/.

### Dependent variables

Two binary dependent variables indicate the presence or absence of single or multiple NCDs (multimorbidity). Questions asked whether individuals had ever been diagnosed or received treatment for arthritis, diabetes, angina, stroke, asthma, depression or chronic lung disease. Information was captured in two ways. Self-reported responses established the presence of diabetes, stroke and chronic lung disease. Symptom-based algorithms, using responses to a number of questions, were used to identify the presence of arthritis, angina, asthma and depression [59, 60]. Hypertension was considered present if the mean of the last two blood pressure measurements was ≥ 140 mmHg (systolic) or ≥ 90 Hg (diastolic) or if respondents reported current treatment with antihypertensive medications [61]. Single morbidity was identified for respondents who had only one of the eight chronic conditions and multimorbidity was identified where respondents had two or more of the eight specified chronic conditions.

### Socioeconomic index

Socioeconomic inequality is measured using an index, or score, derived from household ownership of durable goods (chairs, tables, cars, television, fixed and mobile telephone, bucket or washing machine, or access to electricity), dwelling characteristics (type of floors, walls, and cooking stove), and access to services such as improved water, sanitation, and cooking fuel. The results were recoded into dichotomous variables taking the value of 0 if the household did not possess or have access to the good or service, and 1 if it did. Using a Bayesian post-estimation (empirical Bayes) method, households were arranged on the asset ladder from the poorest to the wealthiest. Raw continuous wealth estimates were transformed into quintiles in the final step. [62]. This is a preferred approach where reliable data about income are difficult and expensive to collect and much of the income earned is non-monetary, or received through transitory or informal employment [63].

### Determinants

This selection of determinants was informed by the literature describing the social, demographic and behavioural correlates of NCDs [5, 8, 9, 22, 33, 64, 65]. Social and demographic determinants are sex, age, marital status, residence, education and work status. Sex was male or female. Age is expressed as: 50–59 years; 60–69 years; 70–79 years or 80+ years. The individual questionnaire asked respondents whether they were never married, currently married, cohabiting, separated/dayivorced or widowed. For this analysis marital status is categorized into three groups: married or cohabiting versus unmarried versus widowed or separated. Residence is categorized according to either urban or rural location. Education and work status variables are also included. The education variable groups respondents according to whether they had: no schooling; less than six years (of schooling); completed primary; completed secondary; completed high school, or completed university or college. The work status variable is categorized into three groups: never worked versus currently working versus currently not working. Using answers to questions on work history in the individual questionnaire, respondents were first divided into two groups, those who ever worked and those who never worked. Individuals who reported having ever worked were further divided into those who were currently working and those who were currently not working.

The following health behavioural factors are included: Body Mass Index (BMI), smoking, alcohol use, fruit and vegetable intake and physical activity. The BMI variable is categorized as underweight if BMI ≤ 18.4, normal if BMI 18.5–24.9, overweight if BMI 25–29.9, and obese if BMI ≥30+. Respondents were asked whether they: had ever smoked tobacco; currently smoked tobacco and smoked daily. Information from responses to these questions was used to derive the smoking variable categorized as: non-smoker; former smoker; current smoker and current daily smoker. Alcohol use is binary: drinkers versus non-drinkers. Fruit and vegetable intake is categorized as adequate (≥5 portions of fruit or vegetable servings per day) and inadequate (≤4 portions consumed daily) [66]. The global physical activity questionnaire [67] is used to assess the intensity, duration and frequency of physical activity during work, transport and leisure time. Respondents are categorized into high, moderate, and low physical activity groups based on the reported time spent in physical activity during a typical week, the number of days of physical activity per week and the intensity of the activity.

The socioeconomic index scores are also converted into quintiles generating ordered categories of household wealth from the poorest (quintile 1) to the richest (quintile 5). These wealth quintiles are included as determinants in the regression-based decomposition analyses described below.

### Statistical analysis

#### Descriptive statistics

The study populations in China and Ghana are described by the determinants and NCD morbidity categories. (Table 1). Numbers of observations and proportions (including survey sampling weights) are reported.

**Table 1:** Characteristics of respondents stratified by NCDs, adults aged 50+, China (2008–2010) and Ghana (2008–2009), SAGE Wave 1

Characteristics	China (*n* = 11,814)			Ghana (*n* = 4,050)		
	No morbidity (*n* = 3,018)	Single Morbidity (*n* = 5,287)	Multimorbidity (*n* = 3,509)	No Morbidity (*n* = 1,000)	Single Morbidity (*n* = 1,828)	Multimorbidity (*n* = 1,222)
Overall rates	25.5%	44.8%	29.7%	24.7%	45.1%	30.2%
Sex						
Male	1,485 (27.19)	2,473 (46.98)	1,524 (25.83)	621 (27.41)	960 (46.88)	537 (25.71)
Female	1,533 (23.15)	2,814 (45.20)	1,985 (31.65)	379 (20.06)	868 (45.66)	685 (34.28)
Age						
50-59	1,786 (32.77)	2,395 (47.79)	1,005 (19.44)	452 (26.25)	764 (48.37)	394 (25.38)
60-69	775 (21.20)	1,594 (46.08)	1,190 (32.72)	264 (22.34)	511 (46.40)	362 (31.26)
70-79	380 (16.23)	1,037 (42.29)	1,050 (41.48)	191 (21.63)	398 (45.28)	321 (33.10)
80+	77 (12.43)	261 (44.33)	264 (43.24)	93 (24.05)	155 (39.67)	145 (36.29)
Residence						
Urban	1,461 (25.66)	2,341 (42.28)	2,025 (32.06)	309 (18.95)	795 (48.74)	541 (32.31)
Rural	1,557 (24.67)	2,946 (49.39)	1,484 (25.94)	691 (27.34)	1,033 (44.62)	681 (28.04)
Marital Status						
Married/Cohabiting	2,643 (26.31)	4,408 (45.96)	2,802 (27.73)	660 (26.80)	1,061 (47.72)	587 (25.48)
Unmarried	26 (24.22)	52 (52.44)	23 (23.34)	16 (44.59)	16 (30.17)	14 (25.24)
Widow/Separated	349 (17.84)	827 (46.33)	684 (35.82)	324 (18.90)	751 (44.66)	621 (36.43)
Wealth						
1 poorest	539 (21.60)	1,085 (48.39)	740 (30.01)	259 (30.19)	358 (46.61)	188 (23.20)
2	610 (24.87)	1,058 (44.31)	703 (30.83)	213 (26.62)	333 (42.20)	252 (31.18)
3	607 (25.35)	987 (44.08)	762 (30.56)	184 (23.27)	361 (44.86)	260 (31.87)
4	626 (24.72)	1,117 (47.11)	713 (28.16)	194 (22.03)	366 (46.00)	274 (31.97)
5 richest	636 (28.40)	1,040 (46.62)	591 (24.98)	150 (18.65)	410 (51.38)	248 (29.97)
Educational level						
No school	567 (20.18)	1,346 (46.01)	960 (33.81)	566 (25.08)	953 (43.51)	693 (31.41)
< 6 years	536 (23.87)	1,012 (48.58)	618 (27.54)	92 (20.50)	184 (47.20)	137 (32.30)
Primary	633 (25.98)	1,043 (46.39)	687 (27.63)	111 (26.66)	224 (50.36)	105 (22.98)
Secondary	667 (26.16)	1,065 (47.07)	673 (26.77)	51 (26.13)	69 (46.95)	45 (26.92)
High School	484 (32.39)	608 (41.53)	400 (26.09)	151 (20.75)	323 (49.91)	201 (29.34)
University/College	131 (28.35)	213 (41.03)	171 (30.63)	29 (21.27)	75 (54.16)	41 (24.57)
Work Status						
Never Worked	345 (25.57)	550 (44.92)	338 (29.51)	21 (29.21)	25 (52.76)	10 (18.02)
Currently working	1,437 (28.56)	2,280 (48.98)	1,043 (22.46)	760 (25.86)	1,341 (47.93)	748 (26.21)
Currently not working	1,236 (21.83)	2,457 (43.53)	2,128 (34.65)	219 (18.81)	462 (41.87)	464 (39.32)
Body Mass Index						
Underweight	198 (39.19)	208 (38.80)	122 (22.00)	190 (32.10)	249 (41.59)	163 (26.31)
Normal	2,153 (29.35)	3,210 (45.00)	1,948 (25.66)	645 (27.12)	997 (45.06)	652 (27.82)
Overweight	591 (17.42)	1,592 (49.43)	1,157 (33.15)	118 (15.26)	402 (50.94)	255 (33.80)
Obese	76 (11.18)	277 (45.17)	282 (43.66)	47 (11.43)	180 (50.89)	152 (37.68)
Physical Activity						
High	1,375 (27.35)	2,282 (48.70)	1,206 (23.95)	678 (25.33)	1,177 (46.41)	743 (28.26)
Moderate	833 (24.37)	1,434 (44.62)	1,078 (31.01)	115 (22.73)	224 (47.24)	162 (30.03)
Low	810 (22.14)	1,571 (43.08)	1,225 (34.78)	207 (20.89)	427 (45.54)	317 (33.56)
Smoking						
Non Smoker	1,925 (23.48)	3,500 (45.85)	2,418 (30.67)	696 (22.97)	1,371 (46.76)	937 (30.27)
Former Smoker	130 (19.11)	293 (44.01)	314 (36.88)	122 (21.27)	259 (48.61)	162 (30.12)
Current Smoker	89 (28.82)	133 (43.39)	84 (27.78)	31 (25.58)	45 (42.97)	34 (31.45)
Current Daily	874 (30.09)	1,361 (47.37)	693 (22.54)	151 (37.02)	153 (38.98)	89 (24.00)
Alcohol Drinking						
No	2,057 (24.96)	3,579 (44.94)	2,492 (30.10)	386 (22.39)	754 (45.90)	528 (31.71)
Yes	961 (25.47)	1,708 (48.24)	1,017 (26.29)	614 (25.02)	1,074 (46.59)	694 (28.39)
Fruit/Veg Intake						
Adequate	2,666 (25.12)	4,647 (46.53)	3,008 (28.35)	273 (21.76)	657 (55.20)	290 (23.04)
Inadequate	352 (25.25)	640 (42.13)	501 (32.62)	727 (24.91)	1,171 (42.21)	932 (32.88)

Actual numbers of observations. Proportions in brackets sum across rows and include survey sampling weights

#### Concentration index

There are a number of methods used to measure socioeconomic inequalities in health. A common approach involves comparing two different groups (so defined by a socioeconomic measure such as wealth) on the basis of a health measure, such as morbidity or mortality. Interpretation is based on the rate ratio or the rate difference between the health measure of the lowest versus the highest socioeconomic group. When percentiles are used, the ratio or difference often refers to quintiles. Although relatively easy to construct and interpret, the rate ratio and rate difference methods mask the extent of the inequality between the two socioeconomic groups.

In their seminal paper Wagstaff et al. [68] proposed that a suitable quantitative measure must meet three requirements which are that the measure should: 1) reflect the socioeconomic dimension of the (ill) health inequality; 2) encompass the experience of the entire population, and 3) be sensitive to changes in rank across socioeconomic groups. The conclusions drawn by Wagstaff and colleagues was that only the concentration index (CI), the slope index of inequality and the relative index of inequality meet these requirements, although it has since been argued that there are other measures which deserve consideration [69].

The CI quantifies the extent of an inequality. This study uses the CI to measure overall socioeconomic inequalities in single and multimorbidity. Individuals with the highest score for household wealth (i.e. the richest) were placed at the top of the ranked distribution, and those with the lowest score (i.e. the poorest) at the bottom. The CI measures whether the health variable (single and multimorbidity) is concentrated among the rich or the poor.

When the health variable is non-binary, the CI ranges from 1 to −1 with negative values indicating concentration of the (ill) health inequality among the poor, and positive values indicating concentration of the (ill) health inequality among the rich. A score of 0 indicates non inequality meaning that, on average, positive and negative effects cancel out across the distribution. In the case of a binary heath measure the CI ranges from μ – 1 (minimum) to 1 − μ (maximum) where μ denotes the mean of the outcome variable [70].

The computation of the CI is based on the approach proposed by Kakwani, Wagstaff, and van Doorslaer [71]:

1 $$ C = \frac{2}{ n\mu}\ {\displaystyle \sum_{i=1}^n}{y}_i{R}_i-1 $$

Where C is the CI, n is the number of observations (or individuals), *y*_*i*_ is the health outcome of individual *i*, μ is the overall mean of y, and *R*_*i*_ is the rank of individual *i* in the socioeconomic distribution moving from the poorest to the richest.

Because the two NCD morbidity outcomes are binary (1,0) a normalization process was applied. This was achieved by multiplying the CI by $\frac{1}{1-\boldsymbol{\mu}}$ in which, the minimum and maximum values of the CI were determined by the mean of the binary variable [70].

The CI is a summary measure of socioeconomic inequality for which there are many possible determinants. These determinants give additional information about the source of the (ill) health inequality [72].

#### Decomposition of the CI

The decomposition method quantifies the extent to which the determinants individually contribute to the overall inequality. The CI can be expressed as the sum of contributions made by determinants (such as social, demographic and behavioural factors) together with an unexplained residual component [72, 73]. The following expresses the linear additive relationship between the health outcome variable *y*_*i*_ the intercept *α*, the relative contributions of the determinants *x*_*ki*_ and the residual error ε_*i*_.

2 $$ {y}_{i = \alpha +{\displaystyle \sum_k}\ {\beta}_k{x}_{k i}+{\varepsilon}_i} $$

The impact that each determinant has on the health variable can be measured using a regression-based approach. When the health variable is binary, the probit model, fitted by the maximum likelihood method, is a suitable method.

The linear approximation of a non-linear relationship using the probit model is based on the following equation:

3 $$ {y}_{i = {\alpha}^m+{\displaystyle \sum_k}{\beta_k}^m{x}_{k i}+{u}_i} $$

where *u*_*i*_ denotes the error produced by the linear approximation to obtain marginal effects (*β*_*k*_^*m*^) which give the change in predicted probability associated with a one unit change in the determinant. Positive signs indicate positive likelihood of the (ill) health outcome and the reverse is true for negative associations. The larger the absolute value of a marginal effect, the more substantial the association. Marginal effects and weighted proportions are reported separately for each of the determinants (Table 2).

**Table 2:** Weighted proportions and marginal effects of determinants of single and multiple NCD morbidity, adults aged 50+, China (2008–2010) and Ghana (2008–2009), SAGE Wave

Determinants	China				Ghana			
	Single Morbidity		Multimorbidity		Single Morbidity		Multimorbidity	
	Weighted Proportion	Marginal Effect	Weighted Proportion	Marginal Effect	Weighted Proportion	Marginal Effect	Weighted Proportion	Marginal Effect
Sex								
Male		Base		Base		Base		Base
Female	0.488	0.0018	0.517	0.0479	0.444	0.0396	0.480	0.0766*
Age								
50-59		Base		Base		Base		Base
60-69	0.304	0.0881***	0.322	0.2145***	0.269	0.0408	0.275	0.0732*
70-79	0.152	0.1279***	0.199	0.3076***	0.215	0.0524	0.230	0.0793*
80+	0.033	0.1755***	0.043	0.3577***	0.086	0.0190	0.107	0.0998*
Residence								
Urban		Base		Base		Base		Base
Rural	0.555	0.0787***	0.501	0.0385	0.607	−0.0423	0.611	−0.0349
Marital Status								
Married/Cohabit		Base		Base		Base		Base
Unmarried	0.011	0.0330	0.009	−0.0120	0.013	−0.2216*	0.016	−0.1001
Widow/separated	0.123	0.0486**	0.136	0.0297	0.355	0.0483	0.405	0.1201***
Educational level								
No school		Base		Base		Base		Base
< 6 years	0.199	−0.0017	0.186	−0.0036	0.101	0.0581	0.103	0.0558
Primary	0.220	−0.0175	0.216	−0.0187	0.121	0.0070	0.102	−0.0769
Secondary	0.206	0.0074	0.197	0.0103	0.042	−0.0138	0.040	−0.0580
High School	0.123	−0.0755**	0.128	−0.0688*	0.175	0.0528	0.162	0.0584
University/College	0.039	−0.0647*	0.044	−0.0501	0.040	0.0364	0.032	−0.0260
Wealth								
Q5 richest		Base		Base		Base		Base
Q4	0.238	0.0180	0.232	0.0727***	0.203	−0.0151	0.211	0.0066
Q3	0.200	−0.0186	0.212	0.0685**	0.198	−0.0072	0.209	0.0251
Q2	0.181	−0.0113	0.193	0.0887**	0.187	−0.0445	0.206	−0.0279
Q1 poorest	0.162	0.0283	0.157	0.0978***	0.199	−0.0221	0.181	−0.1131*
Work Status								
Never Worked		Base		Base		Base		Base
Currently working	0.488	0.0243	0.424	0.0174	0.737	−0.0255	0.680	0.1649
Currently not working	0.435	0.0605*	0.497	0.0957***	0.244	0.0013	0.306	0.3074**
Body Mass Index								
Underweight		Base		Base		Base		Base
Normal	0.629	0.1541***	0.615	0.1765***	0.567	0.0528	0.564	0.0742*
Overweight	0.280	0.2967***	0.280	0.3771***	0.188	0.1800***	0.182	0.2469***
Obese	0.046	0.3582***	0.059	0.5082***	0.089	0.2164***	0.092	0.3181***
Physical Activity								
High		Base		Base		Base		Base
Moderate	0.261	0.0090	0.276	0.0498**	0.123	−0.0196	0.122	−0.0436
Low	0.249	0.0022	0.287	0.0804***	0.232	−0.0153	0.249	−0.0373
Smoking								
Non Smoker		Base		Base		Base		Base
Former Smoker	0.059	−0.0023	0.069	0.0913**	0.141	0.0582*	0.136	0.0886*
Current Smoker	0.026	−0.0801**	0.027	−0.0552	0.025	−0.0128	0.027	0.0450
Current Daily	0.296	−0.0427*	0.266	−0.0491	0.087	−0.0623	0.091	−0.0569
Alcohol Drinking								
No		Base		Base		Base		Base
Yes	0.356	0.0437**	0.330	0.0465*	0.593	0.0058	0.579	−0.0144
Fruit/Veg Intake								
Adequate		Base		Base		Base		Base
Inadequate	0.097	−0.0328	0.110	0.0053	0.655	−0.0694**	0.737	0.1063***

Marginal effects that differ significantly from zero (at p ≤ 0.05) are in bold typeface, * *p* ≤ 0.05, ** *p* ≤ 0.01, *** *p* ≤ 0.001

Given the relationship between *y*_*i*_ and *x*_*ki*_ in equation (2), the CI for y can be re-written as follows:

4 $$ \mathrm{C}\mathrm{I}={\displaystyle \sum_k}\left(\frac{\beta_{\kappa\ {\overline{x}}_k}}{\mu}\right)\ {\mathrm{CI}}_{k + }\ \frac{{\mathrm{GCI}}_{\upvarepsilon}}{\mu} $$

Where μ is the mean of y ${\overline{x}}_k$ is the mean of *x*_*k*_ CI_*k*_ is the CI of *x*_*k*_ and GCI_ε_ isthe generalized CI for the error term. The first term is the deterministic or “explained” component which shows how the CI is explained by systematic variation in the determinants across the socioeconomic distribution. Conversely, the second term is the “unexplained” component because it cannot be explained by systematic variation in the determinants.

The explained component gives further information about the impact that each determinant has on the (ill) health outcome $\left(\frac{\beta_{\kappa\ {\overline{x}}_k}}{\mu}\right)$ and the degree of unequal distribution in each of the determinants across the income groups (CI_k_). If the CI shows that (ill) health is more highly concentrated among the poor, the inequality can arise because a determinant (e.g. rural residence) is more prevalent among people of lower SES and associated with a higher probability of (ill) health [73].

The CI is based upon the relationship between the ranked socioeconomic index and the health outcome in the presence of the determinants. Individuals in the same wealth quintiles may have different ranking on the socioeconomic index. The inclusion of the wealth quintiles variable as a determinant gives information regarding the absolute and relative contributions of wealth (expressed in quintiles) to the overall CI, while controlling for all other determinants [74].

The absolute contribution of each determinant is calculated by multiplying the elasticity. $\left(\frac{\beta_{\kappa\ {\overline{x}}_k}}{\mu}\right)$ of each determinant by the normalised CI for each determinant, CI_k_. The percentage contribution is calculated by dividing the absolute contribution by the summary CIs (Table 3).

**Table 3:** Overall CIs for single and multiple NCD morbidity, adults aged 50+, China (2008–2010) and Ghana (2008–2009), SAGE Wave 1

	China (*n* = 11,814)		Ghana (*n* = 4,050)	
	Dependent variable		Dependent variable	
	Single morbidity	Multimorbidity	Single morbidity	Multimorbidity
Proportion	0.6471	0.5339	0.6594	0.5546
Concentration Index	−0.0365	−0.0801	0.1182	0.1439
95% Confidence Interval	(−0.0689,-0.0040)	(−0.1233,-0.0368)	(0.0697,0.1668)	(0.0794,0.2083)
Subpopulation sample size	8305	6527	2828	2222

For the unexplained component, the CIs for the error terms arecalculated by taking the difference between the CIs for the (ill) health variable and the sum of the contributions by the determinants. Tables 4 and 5 show decompositions of the CIs for China and Ghana respectively.

**Table 4:** Decomposition of CIs of single and multiple NCD morbidity, adults aged 50+, China (2008–2010), SAGE Wave 1

Determinants	Concentration index (C_K_)	Single Morbidity			Multimorbidity		
		Elasticity	Contribution to C_K_ -0.0365	% Contribution	Elasticity	Contribution to C_K_ -0.0801	% Contribution
Wealth							
Q5 richest Base							
Q4	0.3471	0.0066	0.0023	−6.31	0.0315	0.0109	−13.65
Q3	−0.0936	−0.0057	0.0005	−1.47	0.0272	−0.0025	3.18
Q2	−0.4848	−0.0032	0.0015	−4.20	0.0320	−0.0155	19.39
Q1 poorest	−0.8357	0.0071	−0.0059	16.18	0.0288	−0.0241	30.07
Total			−0.0015	4.20		−0.0312	38.98
Sex							
Male Base							
Female	0.0036	0.0013	0.0000	−0.01	0.0463	0.0002	−0.21
Age							
50-59 Base							
60-69	−0.0352	0.0414	−0.0015	4.01	0.1294	−0.0046	5.69
70-79	−0.1260	0.0301	−0.0038	10.42	0.1144	−0.0144	18.00
80+	−0.2470	0.0090	−0.0022	6.08	0.0287	−0.0071	8.84
Total			−0.0075	20.51		−0.0261	32.54
Residence							
Urban Base							
Rural	−0.1966	0.0675	−0.0133	36.41	0.0361	−0.0071	8.86
Marital Status							
Married/Cohabit Base							
Unmarried	−0.4341	0.0006	−0.0002	0.67	−0.0002	0.0001	−0.11
Widow/separate	−0.2435	0.0092	−0.0023	6.17	0.0076	−0.0018	2.30
Total			−0.0025	6.85		−0.0018	2.19
Education level							
No school Base							
< 6 years	−0.1044	−0.0005	0.000	−0.15	−0.0013	0.0001	−0.16
Primary	0.0334	−0.0059	0.000	0.54	−0.0075	−0.0003	0.32
Secondary	0.1424	0.0024	0.000	−0.92	0.0038	0.0005	−0.68
High School	0.3187	−0.0143	−0.005	12.53	−0.0166	−0.0053	6.59
University	0.4871	−0.0039	−0.002	5.26	−0.0041	−0.0020	2.52
Total			−0.0063	17.27		−0.0069	8.59
Work Status							
Never Worked Base							
Currently working	−0.0857	0.0183	−0.0016	4.31	0.0144	−0.0012	1.54
Currently not working	0.1208	0.0407	0.0049	−13.48	0.0891	0.0108	−13.43
Total			0.0033	−9.17		0.0095	−11.89
Body Mass Index							
Underweight Base							
Normal	−0.0435	0.1499	−0.0065	17.87	0.2032	−0.0088	11.03
Overweight	0.1041	0.1284	0.0134	−36.66	0.1976	0.0206	−25.69
Obese	0.0851	0.0255	0.0022	−5.94	0.0563	0.0048	−5.98
Total			0.0090	−24.73		0.0165	−20.63
Physical Activity							
High Base							
Moderate	0.0743	0.0036	0.0003	−0.74	0.0258	0.0019	−2.39
Low	0.0237	0.0008	0.0000	−0.06	0.0432	0.0010	−1.28
Total			0.0003	−0.80		0.0029	−3.67
Smoking							
Non Smoker Base							
Former smoker	0.0438	−0.0002	0.0000	0.02	0.0117	0.0005	−0.64
Current smoker	−0.0901	−0.0032	0.0003	−0.80	−0.0028	0.0003	−0.31
Current daily	−0.0556	−0.0196	0.0011	−2.98	−0.0244	0.0014	−1.70
Total			0.0014	−3.76		0.0021	−2.65
Alcohol Drinking							
No Base							
Yes	−0.0069	0.0240	−0.0002	0.46	0.0288	−0.0002	0.25
Fruit/Veg Intake							
Adequate Base							
Inadequate	−0.1760	−0.0049	0.0009	−2.38	0.0011	−0.0002	0.24
Total			−0.0163			−0.0421	
Residual			−0.0201			−0.0380

**Table 5:** Decomposition of CIs of single and multiple NCD morbidity, adults aged 50+, Ghana (2008–2009), SAGE Wave 1

Determinants	Concentration index (C_K_)	Single Morbidity			Multimorbidity		
		Elasticity	Contribution to C_K_ 0.1182	%Contribution	Elasticity	Contribution to C_K_ 0.1439	%Contribution
Wealth							
Q5 richest Base							
Q4	0.3633	0.0025	0.0009	0.63	−0.0047	−0.0017	−1.43
Q3	−0.0504	0.0095	−0.0005	−0.33	−0.0021	0.0001	0.09
Q2	−0.4454	−0.0103	0.0046	3.20	−0.0126	0.0056	4.76
Q1 poorest	−0.8183	−0.0368	0.0301	20.95	−0.0067	0.0055	4.62
Total			0.0352	24.45		0.0095	8.04
Sex							
Male Base							
Female	−0.0680	0.0664	−0.0045	−3.14	0.0267	−0.0018	−1.54
Age							
50-59 Base							
60-69	−0.0134	0.0363	−0.0005	−0.34	0.0167	−0.0002	−0.19
70-79	−0.0461	0.0329	−0.0015	−1.05	0.0171	−0.0008	−0.67
80+	−0.0783	0.0193	−0.0015	−1.05	0.0025	−0.0002	−0.16
Total			−0.0035	−2.44		−0.0012	−1.02
Residence							
Urban Base							
Rural	−0.2005	−0.0385	0.0077	5.36	−0.0389	0.0078	6.60
Marital Status							
Married/Cohabit Base							
Unmarried	−0.1522	−0.0029	0.0004	0.31	−0.0045	0.0007	0.58
Widow/separate	−0.1210	0.0876	−0.0106	−7.37	0.0261	−0.0032	−2.67
Total			−0.0102	−7.06		−0.0025	−2.09
Education level							
No school Base							
< 6 years	−0.0120	0.0104	−0.0001	−0.09	0.0089	−0.0001	−0.09
Primary	0.1090	−0.0141	−0.0015	−1.07	0.0013	0.0001	0.12
Secondary	0.4413	−0.0042	−0.0019	−1.29	−0.0009	−0.0004	−0.33
High School	0.2554	0.0171	0.0044	3.03	0.0140	0.0036	3.03
University	0.6088	−0.0015	−0.0009	−0.63	0.0022	0.0013	1.14
Total			−0.0001	−0.04		0.0046	3.86
Work Status							
Never Worked Base							
Currently working	−0.0162	0.2023	−0.0033	−2.28	−0.0285	0.0005	0.39
Currently not working	0.0448	0.1694	0.0076	5.27	0.0005	0.0000	0.02
Total			0.0043	3.00		0.0005	0.41
Body Mass Index							
Underweight Base							
Normal	−0.0768	0.0755	−0.0058	−4.03	0.0454	−0.0035	−2.95
Overweight	0.2195	0.0812	0.0178	12.39	0.0514	0.0113	9.54
Obese	0.3873	0.0526	0.0204	14.16	0.0292	0.0113	9.57
Total			0.0324	22.51		0.0191	16.16
Physical Activity							
High Base							
Moderate	0.1012	−0.0096	−0.0010	−0.67	−0.0037	−0.0004	−0.31
Low	0.1698	−0.0167	−0.0028	−1.98	−0.0054	−0.0009	−0.77
Total			−0.0038	−2.65		−0.0013	−1.09
Smoking							
Non Smoker Base							
Former smoker	0.0677	0.0217	0.0015	1.02	0.0125	0.0008	0.71
Current smoker	−0.0387	0.0022	−0.0001	−0.06	−0.0005	0.0000	0.02
Current daily	−0.3713	−0.0093	0.0035	2.40	−0.0082	0.0030	2.57
Total			0.0048	3.37		0.0039	3.30
Alcohol Drinking							
No Base							
Yes	−0.0223	−0.0150	0.0003	0.23	0.0052	−0.0001	−0.10
Fruit/Veg Intake							
Adequate Base							
Inadequate	−0.0309	0.1413	−0.0044	−3.03	−0.0689	0.0021	1.80
Total			0.0584			0.0406	
Residual			0.0855			0.0776	

STATA Version 13 (StataCorp, 2013) and Miscrosoft Excel were used for statistical analyses. Analyses include WHO-SAGE country weights. The SVY command in STATA was used to produce nationally representative estimates and the term [weight] was included in the commands used to derive and decompose the CIs.

#### Study sample

The available samples of individual respondents aged 50 and over who completed the WHO-SAGE individual questionnaires were 13,177 in China and 4,305 in Ghana. The final study samples of 11,814 in China and 4,050 in Ghana were established after removing 1,363 records in China and 255 in Ghana because of missing data on the study variables.

## Results

### Descriptive statistics

Cleaned and complete data from 11,814 individuals in China and 4,050 individuals in Ghana were available for analysis. (Table 1). In China 46.4% (*n* = 5,482) of respondents were male compared with 52.3% (*n* = 2,118) in Ghana. Respondents are stratified according to NCD morbidity: no NCDs versus one NCD versus > = 2 NCDs, defined here as multimorbidity. In China, 25.5% of respondents (*n* = 3,018) were identified as having no NCDs, 44.8% were identified as having one NCD (*n* = 5,287) and 29.7% were identified as having multiple NCDs (*n* = 3,509) compared with 24.7%, 45.1% and 30.2% respectively in Ghana. About 25.6% of urban residents in China had no identified NCDs compared with 19.0% in Ghana. Of respondents in China in the richest wealth quintile, about 28.4% had no identified NCDs compared with 18.7% in Ghana.

The wealth quintiles indicate crude socioeconomic gradients in morbidity. In China, morbidity is more prevalent in the poor. Compared with those in the richest wealth quintile, people in in the poorest quintile have more single morbidity (48.4% versus 46.6%) and more multimorbidity (30.0% and 25.0%). In Ghana the gradient is the reverse. Morbidity is more prevalent in the rich. Compared with people in the poorest quintile, those in the richest quintile have more single morbidity (51.4% versus 46.6%) and more multimorbidity (30.0% and 23.2%).

### Marginal effects of determinants

Table 2 presents the results of the probit logistic regressions for single and multiple NCD morbidity in China and Ghana. Increasing age and living in a rural area were significantly associated with the probability of reporting single morbidity in China (*p* < =0.001). Being widowed or separated was associated with higher probability of reporting single morbidity in China (*p* < =0.01) whereas being unmarried was associated with lower probability of reporting single morbidity in Ghana (*p* < =0.05). Having completed high school and university/college education were significantly associated with lower probability of reporting single morbidity in China (*p* < =0.01 and *p* < =0.05 respectively). Association between the wealth quintiles and single morbidity was not statistically significant in either country.

In China the probability of multimorbidity was highly significant and positive in association with increasing age (*p* < =0.001), having less wealth (*p* < =0.001), not working (*p* < =0.001), being overweight or obese (*p* < =0.001) and having low physical activity (*p* < =0.001). In Ghana the being widowed or separated was significantly associated with multimorbidity (*p* < =0.001) as was being overweight or obese (*p* < =0.001) and having inadequate fruit and vegetable intake (*p* < =0.001). In Ghana those in the lowest wealth quintile had a lower probability of multimorbidity (*p* < =0.05).

### Overall CIs

Table 3 compares the overall CIs for single and multiple NCD morbidity in China and Ghana. The CIs were statistically different from zero at the 5% level. In China, the prevalence of single and multimorbidity was 64.7% and 53.4%, compared with 65.9% and 55.5% respectively in Ghana. In China the negative CI for both morbidity outcomes indicates that the inequality is significant and more highly concentrated among the poor (single morbidity CI = −0.0365: 95% CI = −0.0689, −0.0040; multimorbidity CI = −0.0801: 95% CI = −0.1233, −0.0368). In Ghana the positive CI for both morbidity outcomes indicates that the inequality is significant and more highly concentrated among the rich (single morbidity CI = 0.1182; 95% CI = 0.0697, 0.1668; multimorbidity CI = 0.1453: 95% CI = 0.0794, 0.2083). In both countries, the inequality was higher for multimorbidity.

### CIs of the determinants

Tables 4 and 5 compare the CI_s_ for each of the determinants (CI_*k*_) in China and Ghana. A positive CI_k_ means that the respondents with the characteristic in question were more highly represented among the rich and vice versa. In China, for example, women were more highly represented among the rich (CI_k_ = 0.0036) and in Ghana women were more highly represented among the poor (CI_k_ = −0.0680).

Yet there were some common social gradients. Older respondents were more concentrated among the poor, notably respondents aged 80 and over: CI_k_ = −0.247 in China and CI_k_ = −0.0783 in Ghana. In both countries, respondents residing in rural areas, unmarried, widowed or separated, and currently working, were more highly concentrated amongst the poor. Overweight and obese respondents and those who reported low or moderate physical activity were more highly concentrated among the rich in both China and Ghana.

### Decomposition of socioeconomic inequality in NCD single and multimorbidity

The decomposition shows the contributions (both positive and negative) to the overall socioeconomic inequality by the individual determinants. A positive contribution means that the combined marginal effect of the determinant and its distribution in respect to wealth increases the socioeconomic inequality in the morbidity outcome. Conversely a negative contribution means that the combined marginal effect of the determinant and its distribution in respect to wealth offsets or decreases the socioeconomic inequality in morbidity [75].

Tables 4 and 5 show the absolute and relative contribution of each determinant to the overall wealth inequality in single and multiple NCD morbidity in China and Ghana. In China, the determinants explained −0.0163 of the overall inequality in single morbidity and −0.0421 of the overall inequality in multimorbidity. The unexplained components (i.e. due to the residuals) contributed −0.0201 and −0.0380 respectively. By contrast, in Ghana, the determinants together explained 0.0406 of the overall inequality in single morbidity and 0.0584 of the overall inequality in multimorbidity. The residuals contributed 0.0776 and 0.0855 respectively. The contributions derive from both the elasticity of ill health with respect to the determinants and the wealth related CI_k_ of each determinant. These contributions are negative for China and positive for Ghana. A positive (negative) contribution means that the overall inequality would be lower (higher) if the determinant in question was not present.

In China, the largest contributor to the overall socioeconomic inequality in single morbidity was rural residence with a positive contribution of 36.4%. This was followed by BMI with a negative contribution of 24.7% meaning that the overall socioeconomic inequality was offset by higher BMI in the wealthy. In Ghana the wealth quintiles were the main contributor to overall inequality in single morbidity with a positive contribution of 24.5% followed by BMI with a positive contribution of 22.5%. The determinants most strongly associated with overall socioeconomic inequality in multimorbidity were wealth (quintiles) and age in China accounting for 39.0% and 32.5% respectively. In Ghana the highest contributions were from BMI (16.2%) and the wealth quintiles (8.0%).

## Discussion

Developing countries are undergoing unprecedented social and economic change in their demographic and epidemiological profiles. Population ageing, resulting from increased life expectancies, economic development and decreased fertility, is occurring in all parts of the world, but developing countries are experiencing the most dramatic transitions. The changes alter the socioeconomic distribution of morbidity in ways that create substantial inequalities and inequities in health and wealth between and within countries. Governments urgently need robust reliable epidemiological data in order to take action to improve the health and wellbeing of their older adult populations [3, 4].

This is the first study of its kind to directly compare and decompose socioeconomic inequality in single and multiple NCD morbidity in adults in China and Ghana. The findings accord with differing trends in economic growth, social change, shifting morbidity burdens and healthcare insurance coverage. In a broader context, the study findings are consistent with trajectories of economic growth and epidemiological transition occurring in the Asian and SSA regions.

In China NCD morbidity (both single and multiple) was more highly concentrated among the poor. This finding can be largely explained by China’s economic and regional development. In December 1978 China introduced a series of economic reforms characterised by deregulation and liberalisation of trade and investment in international markets [51]. These reforms have contributed to economic growth and major improvements in income and living standards in the country. For example, the poverty rate in China fell from 64% in 1981 to 10% in 2004. However China’s post-reform growth has been unequally distributed in favour of the rich in urban areas. Inequalities in China are increasing in terms of income, health, education and geography. Economic disparities have risen between the coast and inland, urban and rural areas, and between and within provinces. Both economic growth and the decentralised fiscal system are contributing factors. Local government bodies in China are responsible for funding health and education services and older adults in poor households in underserved and underfunded areas are particularly vulnerable to poor health outcomes [50–53, 76].

However like many other countries in SSA, Ghana is still a very poor country in which, compared with China, economic development is modest, public health infrastructure is relatively weak and the implementation of universal health coverage is slow. Access to care is skewed towards those who are more advantaged in socioeconomic terms. In this study single and multimorbidity were highly concentrated among the rich in Ghana because people of higher SES have relatively better access to services that diagnose and treat chronic illnesses. In older adults of lower SES, healthcare access is further compromised by barriers such as large distances to suitable affordable facilities, transport costs, lack of information about the need for diagnosis and care [42, 45, 46].

The results can also be explained by behavioural and lifestyle change associated with increasing income. Rapid economic development in China is fuelling urbanisation, affluence and consumerism. The traditional way of life has been supplanted by lower levels of physical activity and unhealthy diets, which are risk factors for NCDs [2, 56]. In China NCD behavioural risk factors are prominent amongst those in poorer socioeconomic circumstances whereas in Ghana these lifestyle behaviours are more common among the relatively affluent. These patterns have been observed globally with respect to country income levels; NCDs are more prevalent among the rich in economically less prosperous countries (e.g. in Ghana) but as economic development increases and incomes rise (e.g. in China) the association is reversed [50, 51, 77].

It is also important to understand the study findings in the context of contrasting disease burdens. China has had success in tackling infectious diseases, and the burden is now mostly due to NCDs [55]. China’s economic success has afforded healthcare infrastructure for costly conditions such as cardiovascular disease, mental health and respiratory conditions although the NCD epidemic is now impacting on further economic growth and development [56]. Ghana, like many other countries in SSA, faces a mixed morbidity burden from infectious and other conditions prevalent in younger age, as well as NCDs. A WHO report on Ghana showed that 53% of all-age mortality was due to communicable, maternal, perinatal and nutritional conditions, while 39% was attributed to NCDs and 8% to injuries 8% [78]. In Ghana health resources and systems remain heavily constrained by prioritised treatments of communicable, maternal, perinatal and nutritional conditions among younger people. Health-seeking behaviours for NCDs in many countries in SSA have not been fully realised although there is a view that this will change as incomes rise and the countries become more prosperous [32, 43, 45, 48].

Health insurance coverage is an important factor in ensuring health utilization occurs equally and fairly and in accord with need [76]. The study’s results highlight differences in the timing and implementation of universal healthcare coverage in China and Ghana. Compared with many other countries, China has made notable progress towards achieving universal healthcare insurance. Between 1978 and 2002 health insurance in China was a benefit bestowed by the Government to employed people under the Urban Employee Basic Medical Insurance Scheme. Beginning in 2002 the Government introduced reforms which extended insurance coverage to vulnerable groups in the population, such as the unemployed, the elderly, students and children. Yet implementation in rural areas has lagged behind urban areas [54].

The decomposition for China showed that wealth, older age and rural residence all contributed to socioeconomic inequality in single and multiple NCD morbidity. These findings are consistent with evidence of inequities in China’s healthcare coverage showing that respondents in some rural areas, those in older age and with less income had a lower chance of being insured [54, 76] and that the health of the elderly is effected by factors such as income and residence [52].

Ghana’s reintroduction of user fees for healthcare, post gaining independence from Britain in 1957, resulted in decreased use of healthcare services, particularly among those on low incomes and in older age. Many people delay seeking treatment and rely on self-medication. Only about a quarter of people in households with at least one adult member aged 50 or over have health insurance, and consequently these households are more likely to experience catastrophic health expenditure and impoverishment [46, 48].

In the decomposition for Ghana, wealth and BMI were main contributors to socioeconomic inequality in single and multiple NCD morbidity. This finding is consistent with other national studies in Ghana showing a positive association between obesity and wealth quintiles whereby higher obesity prevalence is associated with higher wealth. Being overweight or obese is associated with affluence in Ghana and these behaviours are major metabolic risks associated with many NCDs such as diabetes mellitus and hypertension [46, 48].

### Strengths and limitations

To our knowledge, this is the first study to measure socioeconomic inequality in multimorbidity in China and Ghana using the CI, and to decompose socioeconomic inequality by a set of determinants. This study uses a nationally representative data set (SAGE) that allows for comparison with other similar datasets such as that of the Study on Health and Retirement in Europe (SHARE). This study also provides new evidence and information to add to the literature regarding socioeconomic inequalities in the health of older adults in China and Ghana.

This study does not attempt to determine causality, rather it highlights associations. Longitudinal analyses are needed to identify causality. We acknowledge the possibility of reporting bias. Individuals were asked whether they had ever been diagnosed (by a physician) or received treatment for diabetes, stroke and chronic lung disease. Yet LMICs often have limited public health resources and infrastructure; clinical services for the diagnosis and treatment of chronic conditions are not always available and accessible. Self-reported answers to questions asked about diagnosed chronic conditions can therefore underestimate true disease prevalence, although we are unable to say the extent to which this may have occurred. Additionally, information regarding infectious or communicable diseases was not included in these data. Although such diseases are often more prevalent among younger people, we do not know the extent to which they were prevalent in our study sample.

## Conclusions

In China, NCD single and multiple morbidity was concentrated among the poor whereas in Ghana morbidity was more concentrated among the rich. On one level this result suggests that China, rather than Ghana, needs to take more urgent action to tackle unfair inequalities in health and wealth. However the results warrant deeper contextual interpretation as discussed here. The inequalities observed for Ghana are indicative of somewhat exclusive healthcare use by more advantaged older adults.

The country comparison reflects different stages of economic development and social change in China and Ghana that are representative of major global trends - social and economic development as well as demographic and epidemiological transitions. Developing countries face particular challenges because their older adult populations are increasing and this population group is also experiencing age-related ill health and disability. Although economic development can, to some extent, provide a panacea this comes at a cost, as is the case in China. Governments in both counties need to take inter-sectoral actions to address what are major global issues.

## Abbreviations

BMI: Body mass index
CHE: Catastrophic health expenditure
CI: Concentration index
KG_s_: Kilograms
LMICs: Low- and middle-income countries
Mm Hg: Millimetres of mercury
M_s_: Metres
NCDs: Non-communicable diseases
PSUs: Primary sampling units
SAGE: Study on global AGEing and adult health
SES: Socioeconomic status
SHARE: Study on health and retirement in Europeat
SSA: Sub-Saharan Africa
VIF: Variance inflation factor
WHO: World health organization
